# Adaptive Rate-Compatible Non-Binary LDPC Coding Scheme for the B5G Mobile System

**DOI:** 10.3390/s19051067

**Published:** 2019-03-02

**Authors:** Dan-feng Zhao, Hai Tian, Rui Xue

**Affiliations:** College of Information & Communication Engineering, Harbin Engineering University, Harbin 150001, China; zhaodanfeng@hrbeu.edu.cn (D.-f.Z.); xuerui@hrbeu.edu.cn (R.X.)

**Keywords:** B5G, adaptive coding scheme, channel clustering, k-means++ algorithm, rate-compatible LDPC

## Abstract

This paper studies an adaptive coding scheme for B5G (beyond 5th generation) mobile system-enhanced transmission technology. Different from the existing works, the authors develop a class of rate-compatible, non-binary, low-density parity check (RC-NB-LDPC) codes, which expresses the strong connection between the algebra-based and graph-theoretic-based constructions. The constructed codes can not only express rate-compatible (RC) features, but also possess a quasi-cyclic (QC) structure that facilitates the encoding implementation. Further, in order to achieve the code rate-adaptive allocation scheme, the authors propose using the K-means++ clustering algorithm to cluster different channel environments, considering various factors that affect channel characteristics. Finally, in order to present the advantages of the adaptive coding scheme, the authors construct a coding scheme for image transmission. The numerical results demonstrate that the developed code can obtain better waterfall performance in a larger code rate range, which is more suitable for data transmission; the adaptive coding transmission scheme can obtain higher reconstructed image quality compared to the fixed code rate-coding scheme. Moreover, when considering unequal error protection (UEP), the proposed scheme can further improve the reconstructed image quality.

## 1. Introduction

With the advent of the 5G communication era, the demand for wireless communication is still strong, and the increased spectrum and power resources are insufficient. Therefore, new transmission technology needs to be updated to achieve rapid growth of the wireless communication capacity [1,2]. The B5G mobile system performs well in terms of wireless coverage and user experience. To overcome the challenge, one should improve the error-correcting, anti-jamming, and efficient transmission capabilities for B5G. Among the communication technologies for error correction and detection, channel coding can significantly improve the reliability of communication systems and ensure efficient transmission—this has become one of the focuses among the communication technologies [3,4,5,6,7,8,9].

In order to achieve significant improvements with key performance parameters, the channel-coding technique for B5G should have features such as large coding gain, low-complexity, low-latency, high-throughput, and flexible code parameters [10,11]. The conventional single channel-coding schemes can hardly meet all of the requirements of B5G. The B5G system will select different channel-coding schemes for different scenarios and serves to meet the corresponding technical requirements.

For the B5G mobile system, the channel-coding scheme for a high data rate is pursued; however, the wireless channel environment is time-varying and fading. High-data-rate signals are prone to inter-symbol interference (ISI) in frequency-selective fading channels, which affects the overall transmission performance of the system [12]. In order to improve the throughput of the system, the adaptive coding method is proposed [13]. This method dynamically adjusts the coding mode of the transmitted signal according to the channel state information (CSI) fed back from the receiving end, and then finds a balance between the transmission rate and the transmission reliability to improve the throughput of the system. The DVB-S2 standard adopts an adaptive coding modulation (ACM) scheme in which channel coding comprises LDPC codes [14,15]. However, the parameters such as the code length and the rate of LDPC codes in the DVB-S2 standard are fixed, and LDPC codes of different parameters need to design corresponding check matrices, resulting in the consumption of hardware resources.

LDPC codes are currently among the most promising coding techniques to achieve the channel capacities for a wide range of channels. LDPC codes were first proposed by Gallager in 1962 [16], and developed in the late 1990s [17,18]. Since their rediscovery, a great deal of research has been conducted in design, construction, encoding, and decoding algorithms. Many LDPC codes have been adopted as standard codes for current and next-generation communication systems, such as wireless, optical, satellite, deep space, multi-media broadcast (MMB), digital video broadcast (DVB) systems [8,9,10], and so on. This rapid dominance of LDPC codes in applications is due to their capacity-approaching performance, and they can be achieved with practically implementable iterative decoding algorithms. However, further research is needed to better understand the structural properties and performance characteristics of these codes. QC-LDPC codes, a subclass of LDPC codes [11], can be efficiently encoded and decoded by shift register adder accumulator (SRAA) [19,20] and semi-parallel decoding architectures [21,22], respectively. Thus, QC-LDPC codes can greatly reduce the consumption of hardware resources, and solve the complicated problem of implementing the codec from a certain angle.

Rate-compatible LDPC (RC-LDPC) codes are a special set of LDPC codes with different code rates. These LDPC codes have good performance at each rate and can be implemented using the same set of codecs. The communication system using the RC-LDPC code can adjust the code rate according to the current channel condition, so that the communication system can improve the transmission efficiency while satisfying the transmission reliability. In 2002, J Li and KR Narayanan combined the puncturing and extension methods to generate a series of RC-LDPC codes, which expanded the range of dynamic code rates [23]. In 2006, J. Ha selected the location of the puncturing variable node from the perspective of the recovery tree, and proposed a grouping/sorting algorithm to sort the variable nodes according to the size of the recovery tree. The node with the smallest number of layers is used as the puncturing node [24]. In 2007, H.Y Park proposed another perforation measure [25]. The non-greedy criterion puncturing algorithm proposed in [26] shows better performance than the recovery tree-based puncturing algorithm. In [27], the shortening priority of variable nodes is divided according the column weight of each column in the check matrix, and a shortening algorithm suitable for whether irregular or regular quasi-cyclic LDPC codes was proposed.

The traditional ACM technology selects the corresponding modulation and coding schemes according to the threshold calculated by the CSI fed back from the receiver, which depends largely on the assumed channel model [28,29]. In fact, the real channel is not Gaussian-distributed; the amplifier incurs nonlinear effects, etc. Using the traditional ACM algorithm, it is often difficult to meet the frame error rate (FER) requirement [12,30]. With the rise of machine learning algorithms, more simple and effective methods are used to reduce the complexity of the traditional methods [6,31,32]. The clustering algorithm is an important unsupervised learning algorithm in machine learning. Due to its flexibility, it is widely used in various technical fields [33,34].

This paper studies an adaptive coding scheme for the B5G mobile system. We first develop a class of RC-NB-LDPC codes based on masking and puncturing techniques to construct the RC-LDPC codes. The masking technique converts the check matrix of the regular non-binary LDPC (NB-LDPC) code into one of irregular NB-LDPC code, and the puncturing technology constructs RC-NB-LDPC code. Then, we design a rate allocation scheme. Different from the existing works, we propose using the k-means++ clustering algorithm to cluster different channel environments, considering various factors that affect channel characteristics. This method relies on a perfect mathematical model and can learn interactively in the wireless channel. According to the actual error rate performance of the system, the correspondence between the signal-to-noise ratio and the modulation and coding mode is determined, which helps to select a more accurate coding rate. Finally, in order to present the advantages of the adaptive coding scheme in compressed image processing, we construct a coding scheme for image transmission. The numerical results demonstrate that the developed code can obtain better waterfall performance in a larger code rate range, which is more suitable for data transmission; the adaptive coding transmission scheme can obtain higher reconstructed image quality compared to the fixed code rate-coding scheme. Moreover, when considering UEP, the proposed scheme can further improve the reconstructed image quality.

The remainder of this paper is organized as follows. The construction method and notations of RC-NB-LDPC codes are described in Section 2. In Section 3, we propose using the channel-clustering algorithm to cluster different channel parameters. In Section 4, we discuss the BER performance and decoding thresholds of RC-NB-LDPC codes. We also construct an adaptive coding scheme for image transmission to evaluate the performance of reconstructed image quality. We present our conclusions in the final section.

## 2. The Construction of RC-NB-LDPC Codes

In this section, we first briefly review a general matrix-theoretic method for constructing QC-LDPC codes based on superposition. Then, we develop a class of RC-NB-LDPC codes based on masking and puncturing techniques.

### 2.1. QC-LDPC Codes and Superposition Construction

The check matrix H corresponding to a class of quasi-cyclic LDPC codes can be expressed by

(1) $$H=\left[\begin{array}{cccc} I\left(P_{11}\right) & I\left(P_{12}\right) & \begin{array}{ccc} . & . & . \end{array} & I\left(P_{1n}\right)\\ I\left(P_{21}\right) & I\left(P_{22}\right) & \begin{array}{ccc} . & . & . \end{array} & I\left(P_{2n}\right)\\ \begin{array}{c} .\\ .\\ . \end{array} & \begin{array}{c} .\\ .\\ . \end{array} & \begin{array}{ccc} . & . & . \end{array} & \begin{array}{c} .\\ .\\ . \end{array}\\ I\left(P_{m1}\right) & I\left(P_{m2}\right) & \begin{array}{ccc} . & . & . \end{array} & I\left(P_{mn}\right) \end{array}\right]$$

where $I\left(P_{ij}\right)\left(1\leq i\leq m,1\leq j\leq n\right)$ is a b×b-dimensional circulant matrix, and is referred to as the circulant permutation matrix [35]. This matrix can be obtained by rotating each row vector of a b×b-dimensional unit matrix $P_{ij}$ element to the right, and $P_{ij}\in \left\{0,1,2,\cdots ,b-1,\infty \right\}$. Herein, $P_{ij}=0$ means that $I\left(P_{ij}\right)$ is a unit matrix, and $P_{ij}=\infty$ means that $I\left(P_{ij}\right)$ is a zero matrix. For any i,j, replacing the circulant permutation matrix $I\left(P_{ij}\right)$ in the check matrix H by value $P_{ij}$, one can obtain a new matrix P, referred to as the circulation coefficient-matrix [36], which is shown by

(2) $$P=\left[\begin{array}{cccc} P_{11} & P_{12} & \begin{array}{ccc} . & . & . \end{array} & P_{1n}\\ P_{12} & P_{22} & \begin{array}{ccc} . & . & . \end{array} & P_{2n}\\ \begin{array}{c} .\\ .\\ . \end{array} & \begin{array}{c} .\\ .\\ . \end{array} & \begin{array}{ccc} . & . & . \end{array} & \begin{array}{c} .\\ .\\ . \end{array}\\ P_{m1} & P_{m2} & \begin{array}{ccc} . & . & . \end{array} & P_{mn} \end{array}\right]$$

$H_{ij}=0$ when $P_{ij}=\infty$ and $H_{ij}=1$; otherwise, for any i,j, one can obtain the corresponding base matrix $H_{b}$, which is given by

(3) $$H_{b}=\left[\begin{array}{cccc} H_{11} & H_{12} & \begin{array}{ccc} . & . & . \end{array} & H_{1n}\\ H_{12} & H_{22} & \begin{array}{ccc} . & . & . \end{array} & H_{2n}\\ \begin{array}{c} .\\ .\\ . \end{array} & \begin{array}{c} .\\ .\\ . \end{array} & \begin{array}{ccc} . & . & . \end{array} & \begin{array}{c} .\\ .\\ . \end{array}\\ H_{m1} & H_{m2} & \begin{array}{ccc} . & . & . \end{array} & H_{mn} \end{array}\right]$$

where $H_{ij}\in \left\{0.1\right\},\left(1\leq i\leq m,1\leq j\leq n\right)$.

Thus, if we obtain a small m×n base matrix $H_{b}$ and a set of b×b circulant permutation matrices, we can construct an m×n array H of b×b matrices by replacing each 1-entry in $H_{b}$ by a circulant permutation matrix and each 0-entry by a zero matrix. This replacement of entries in $H_{b}$ by the circulant permutation matrices and/or zero matrices expands the matrix $H_{b}$ into a mb×nb sparse matrix H.

### 2.2. The Construction of RC-NB-LDPC Codes

**Figure 1:**
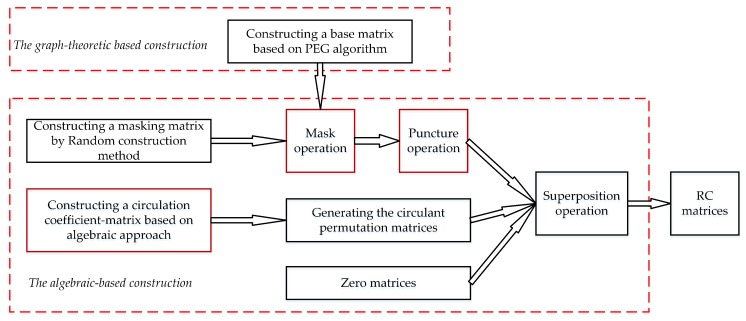
RC-NB-LDPC codes construction process.

Most methods for constructing LDPC codes can be classified into two general categories: the algebraic-based and the graph-theoretic-based constructions [36]. This paper combines these two methods to construct the RC-LDPC codes, as shown in Figure 1.

The best-known graph-theoretic-based construction method is the progressive edge-growth (PEG) algorithm [37,38]. The goal of this algorithm is to construct a graph that adds one edge each time and maximizes the local girth. Therefore, the PEG algorithm is a greedy algorithm for constructing a Tanner graph with a large girth. In this paper, we use the PEG algorithm to construct a small base matrix for future research.

The algebraic-based methods for constructing LDPC codes have been developed using mathematical tools such as finite geometries [39], finite fields [40], and combinatorial designs [41,42]. In general, Algebraic LDPC codes have much lower error-floors than randomly constructed LDPC codes. Most of the algebraic constructions involve several key ingredients including the circulant permutation matrix, masking matrix, and superposition operation [43]. In this paper, the key operations for constructing RC-NB-LDPC codes are described as follows.

• Mask operation

The masking technique can selectively mask some sub-circulant matrixes in order to reduce the density of non-zero elements in the check matrix H, so that fewer small cycles or longer girth could be obtained. Moreover, the performance of the LDPC code is improved. Through selecting a suitable masking matrix, we can obtain any rate code according to the same base matrix.

The masking technique can be described as special matrix product operations [44]. We assume a masking matrix $H_{m}=\left[m_{ij}\right]$ as a binary matrix with size of m×n, then the definition of the product of $H_{m}$ and H is shown as (4).

(4) $$H=H_{m}⊗H_{b}\equiv \left[\begin{array}{cccc} m_{1,1}I\left(P_{11}\right) & m_{1,2}I\left(P_{12}\right) & \begin{array}{ccc} . & . & . \end{array} & m_{1,n}I\left(P_{1n}\right)\\ m_{2,1}I\left(P_{21}\right) & m_{2,2}I\left(P_{22}\right) & \begin{array}{ccc} . & . & . \end{array} & m_{2,n}I\left(P_{2n}\right)\\ \begin{array}{c} .\\ .\\ . \end{array} & \begin{array}{c} .\\ .\\ . \end{array} & \begin{array}{ccc} . & . & . \end{array} & \begin{array}{c} .\\ .\\ . \end{array}\\ m_{m,1}I\left(P_{m1}\right) & m_{J,2}I\left(P_{m2}\right) & \begin{array}{ccc} . & . & . \end{array} & m_{mn}I\left(P_{mn}\right) \end{array}\right]$$

In Equation (4), when $m_{i,j}=1$, $m_{i,j}A_{s_{ij}}=A_{s_{ij}}$ and when $m_{i,j}=0$, $m_{i,j}A_{s_{ij}}=0$. We define $H_{b}$ as the base matrix and H as the masked matrix. If $H_{m}$ and $H_{b}$ are sparse matrices, then H is also a sparse matrix. In addition, sub-matrices in H can be replaced with zero arrays of the same size by using the masking technique, which can reduce the density of non-zero elements in GF(q). Thus, a sparser matrix H degrades the probability of small cycles.

When constructing LDPC codes, if we obtain the degree distributions λ(x) and ρ(x) of the variable node and the check node, then we can obtain the check matrix H. We assume that the corresponding degree distribution of code words with code rate r and code length n are λ(x) and ρ(x); if the masking matrix $H_{m}$ obeys the same degree distribution, the masked check matrix H will also obey the same distribution.

• Puncture operation

The puncturing technique is the most common rate adaptive technique [24,45,46], the implementation process of which first selects a high rate mother code with excellent performance, then punctures the check bit in the code word, thus deleting a number of columns in the check matrix, resulting in a low rate code word. The high rate QC-LDPC code may be used at higher SNR. On the contrary, when the channel environment is poor, a low rate QC-LDPC code may be used, thereby improving the average effectiveness of the communication system. Using the puncturing technique to change the degree distribution of the masking matrix, an adaptive-rate LDPC code can be obtained. Since the dimension of the masking matrix is much smaller than the check matrix of the QC-LDPC code, it can greatly reduce the hardware complexity of the rate-adaptive communication system.

The NB-LDPC code defined on the finite field GF(q) has $q=2^{m}$ elements; the mapping relationship is as follows:

(5) $$F_{q}\to F_{2}^{m}$$

We refer to $\left(x_{0},\ldots ,x_{m-1}\right)\in F_{2}^{m}$ as a binary mapping of the symbol $X\in F_{q}$. The NB-LDPC code can be mapped into binary code for transmission in a channel. Therefore, the NB-LDPC code can be punctured at both the symbol level and bit level.

**Figure 2:**
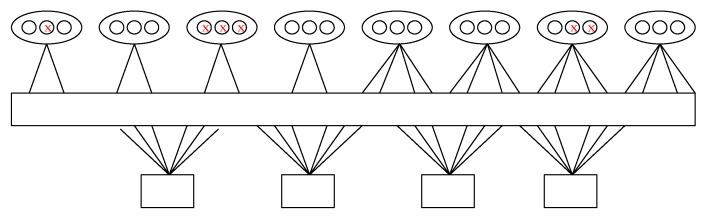
The bit-level puncturing pattern of the NB-LDPC code.

For example, for 8-ary LDPC codes, the degree distribution function is $\lambda \left(x\right)=1/3x+2/3x^{3},$ $\rho \left(x\right)=x^{5}.$ The bit-level puncturing algorithm is intuitively represented by Figure 2.

• The circulation coefficient-matrix is designed using an algebraic-based method

The circulation coefficient-matrix of size m×n is designed using an algebraic-based method, assuming that GF(q) is a finite field containing q elements, α is a primitive of GF(q), and all elements in GF(q) can be represented as a form of α power, i.e., $\alpha^{-\infty },\alpha^{0},\alpha^{1},\ldots ,\alpha^{q-2}$, where $0=\alpha^{-\infty },\alpha^{0}=1$. $S_{1}=\left\{\alpha^{i_{0}},\alpha^{i_{1}},\ldots ,\alpha^{i_{m-1}}\right\}$ and $S_{2}=\left\{\alpha^{j_{0}},\alpha^{j_{1}},\ldots ,\alpha^{j_{n-1}}\right\}$ are two subsets of GF(q), where $i_{k},j_{l}\in \left\{0,1,\ldots ,q-2\right\},0\leq k<m,0\leq l<n$. Based on the closeness of the computation of the finite field, a finite field matrix on GF(q) can be constructed from $S_{1}$ and $S_{2}$.The specific construction method is given by

(6) $$P=\left[\begin{array}{cccc} \alpha^{i_{0}}-\alpha^{j_{0}} & \alpha^{i_{0}}-\alpha^{j_{1}} & \begin{array}{ccc} . & . & . \end{array} & \alpha^{i_{0}}-\alpha^{j_{n-1}}\\ \alpha^{i_{1}}-\alpha^{j_{0}} & \alpha^{i_{1}}-\alpha^{j_{1}} & \begin{array}{ccc} . & . & . \end{array} & \alpha^{i_{1}}-\alpha^{j_{n-1}}\\ \begin{array}{c} .\\ .\\ . \end{array} & \begin{array}{c} .\\ .\\ . \end{array} & \begin{array}{ccc} . & . & . \end{array} & \begin{array}{c} .\\ .\\ . \end{array}\\ \alpha^{i_{m-1}}-\alpha^{j_{0}} & \alpha^{i_{m-1}}-\alpha^{j_{1}} & \begin{array}{ccc} . & . & . \end{array} & \alpha^{i_{m-1}}-\alpha^{j_{n-1}} \end{array}\right]=\left[\begin{array}{cccc} \alpha^{c_{0,0}} & \alpha^{c_{0,1}} & \begin{array}{ccc} . & . & . \end{array} & \alpha^{c_{0,n-1}}\\ \alpha^{c_{1,0}} & \alpha^{c_{1,1}} & \begin{array}{ccc} . & . & . \end{array} & \alpha^{c_{1,n-1}}\\ \begin{array}{c} .\\ .\\ . \end{array} & \begin{array}{c} .\\ .\\ . \end{array} & \begin{array}{ccc} . & . & . \end{array} & \begin{array}{c} .\\ .\\ . \end{array}\\ \alpha^{c_{m-1,0}} & \alpha^{c_{m-1,1}} & \begin{array}{ccc} . & . & . \end{array} & \alpha^{c_{m-1,n-1}} \end{array}\right]$$

where $\alpha^{c_{k,l}}=\alpha^{i_{k}}-\alpha^{j_{l}},0\leq k<m,0\leq l<n$. Matrix P is a circulation coefficient-matrix.

In summary, the steps of constructing the RC-NB-LDPC codes are as follows:

1. Construct a matrix $H_{b}$ of size m×n based on the PEG algorithm as the base matrix.
2. Construct a matrix $H_{m}$ of size m×n based on the random construction as the mask matrix.
3. Mask and puncture the base matrix $H_{b}$.
4. Calculate a circulation coefficient-matrix P of size m×n using an algebraic-based method and generate the circulant permutation matrices.
5. Superposition operation. Replacing the entries in $H_{b}$ by the circulant permutation matrices and/or zero matrices expands the matrix $H_{b}$ into a mb×nb sparse matrix H.

### 2.3. Design and Implementation of the RC-NB-LDPC Code

The check matrix of the RC-NB-LDPC code has a QC structure, so the coding algorithm can adopt the QC-structured fast-coding method. The FPGA implementation based on the QC-structured fast-coding method will be described in detail below.

#### 2.3.1. Matrix of RC-NB-LDPC Codes

**Figure 3:**
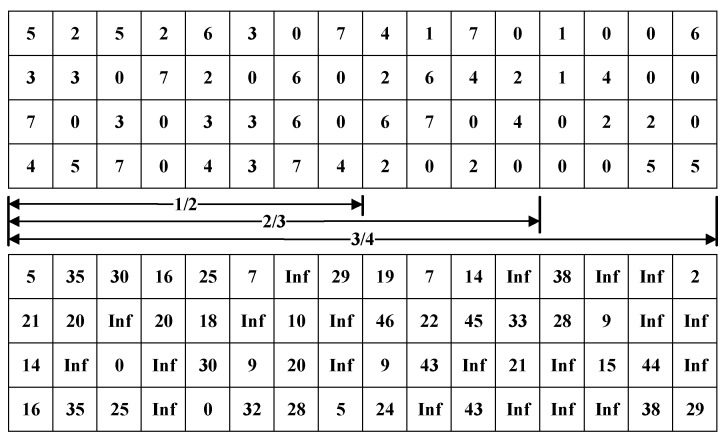
Check matrix H structure of the NB-LDPC codes.

The fast-coding method uses the special structure of the QC-LDPC code for encoding, so the check matrix and the generator matrix need to be set. The structure of the check matrix is shown in Figure 3. The base matrix size is 4×16, and the check matrix is divided into 64 blocks, and the size of each block matrix is 48×48. The numbers in the above matrix represent the element coefficients of the current matrix, and the matrix with a coefficient of 0 is the all-zero matrix. The numbers in the below matrix represent the circulant coefficients of the current matrix, and a coefficient of 0 indicates that the current matrix is not shifted.

**Figure 4:**
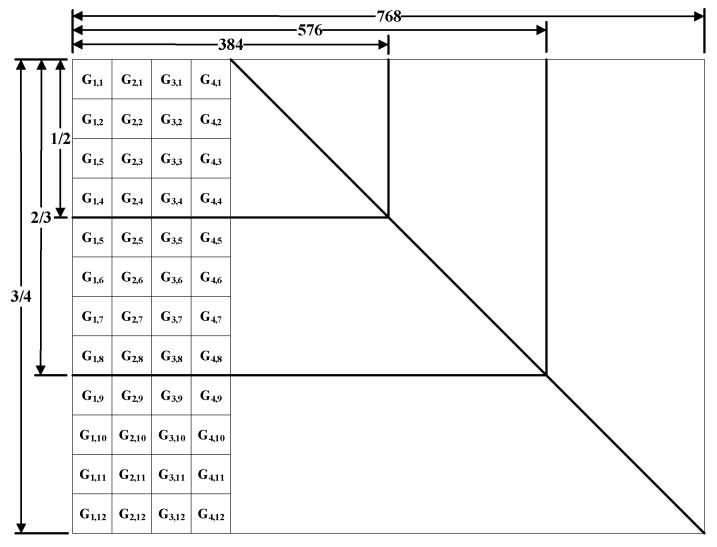
Generation matrix G structure of NB-LDPC codes.

Since the check matrix of the NB-QC-LDPC code has a QC structure, the generator matrix also has a QC structure. The generation matrix is as shown in Figure 4. The right side of the matrix is a unit matrix of size 576×576, and each $G_{i,j}$ on the left side is a circulant of size 48×48. Therefore, the entire matrix can be stored by simply storing the first row of each $G_{i,j}$ matrix, greatly reducing resource usage. The source symbol is multiplied by the matrix of its corresponding code rate to obtain the coding result. Since the right side of the matrix is a unit matrix, it is only necessary to calculate the result of multiplying the source symbol by the $G_{i,j}$, and then stitching the source symbol to obtain the final coding result. The source symbol and $G_{i,j}$ are encoded to obtain a check sequence, wherein $G_{i,j}$ is divided into four columns, which can be encoded simultaneously by four channels, so that the coding efficiency can be improved four-fold.

#### 2.3.2. Encoder Architecture

**Figure 5:**
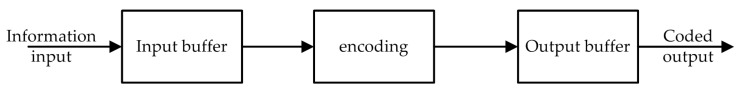
The coding block diagram.

The encoder-coding block diagram is shown in Figure 5. It is divided into three parts. The input buffer part is used to buffer the information transmitted by the upper level and output the data when the encoding part is needed. The second part is the encoding part, which is used to calculate the encoding result and output the calculation result to the output buffer module. The output buffer part is used to store the encoding result and the communication with the downstream module.

**Figure 6:**
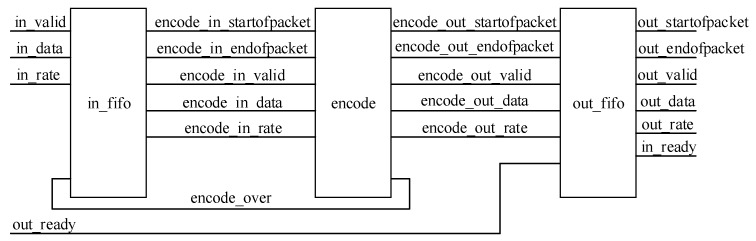
The hardware structure of the NB-QC-LDPC code encoder.

The hardware structure of the NB-QC-LDPC code encoder is shown in Figure 6, which includes an input module, an encoding module, and an output module.

In addition to buffering the source symbol function passed by the previous level, the input module also completes the cross-clock domain operation of the data. Furthermore, according to the currently input code rate information, the source symbol is truncated into a sequence of corresponding lengths, so that the coding module performs coding. Therefore, the input module mainly includes a fifo module and a corresponding control module. When the “encoding_over” signal of the encoding module is high, it indicates that the encoding module has been encoded, and can receive a new source frame.

The coding module is a core module of the NB-LDPC encoder, and its function is to perform a matrix multiplication operation on the source information and the generation matrix in the Galois field to obtain a coding result. The coding module mainly includes an operation module, a RAM storage module and a corresponding control module.

The output module mainly performs the function of storing the encoded result and communicating with the downstream module. The encoding module inputs the encoding result to the output module. When the “out_ready” signal is high, it indicates that the next level has been prepared for reception, and the encoding result may be output; otherwise, the output module temporarily stores the encoding result. When the output module is full, the output “in_ready “signal is turned low, indicating that new data cannot be accepted. Therefore, the output module mainly contains the fifo module and the corresponding control module.

#### 2.3.3. Coding Module

**Figure 7:**
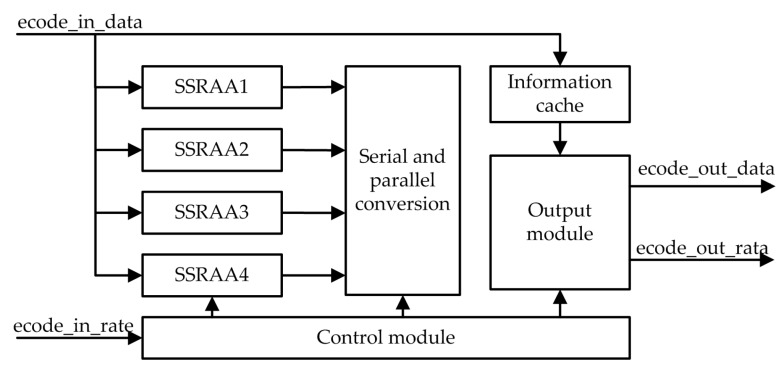
Coding module structure.

The coding module is the core module of the encoder. The block diagram of the structure is shown in Figure 7. It includes the SSRAA (serial shift register accumulator unit) operation module, the source information cache module, the parallel-to-serial conversion module, the output module and the control module.

The SSRAA operation module completes the coding operation process, and the four sets of SSRAA operation modules work in parallel, which improves the coding efficiency. The parallel–serial conversion module converts the verification sequence encoded by the four parallel SSRAA circuits into a serial form for sequential output. The source information cache module is used to store the source sequence. The output module mainly completes the output of the encoded result.

**Figure 8:**
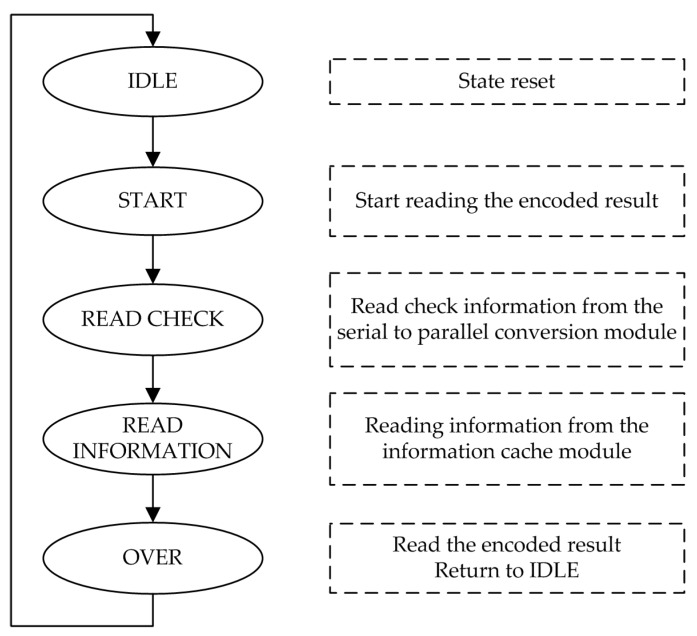
The state machine of the output module.

After the processing of the upper input module, the input data of the encoding module is a piece of truncated source information, and the data length corresponds to the current encoding rate. At the time of data entry, the SSRAA module is also constantly calculating the check sequence. When a piece of information is entered, the SSRAA module also completes the calculation of the check sequence. The output of the encoded result is then performed, and the state machine of the output module is shown in Figure 8.

After the encoding is completed, the state machine jumps from the original IDLE state to the SRTART state and begins reading the encoded result. In the READ_CHECK state, the output module reads the parallel-converted 192-bit check sequence from the parallel-to-serial conversion module and then jumps to the READ_INFORMATION state. In the READ_INFORMATION state, the output module reads the source information of the corresponding length from the information cache module according to the code rate information of the current frame, and completes the output of the encoded result. The state machine then jumps back from the OVER state to the IDLE state, waiting for the encoding of the next frame to complete.

#### 2.3.4. SSRAA Module

**Figure 9:**
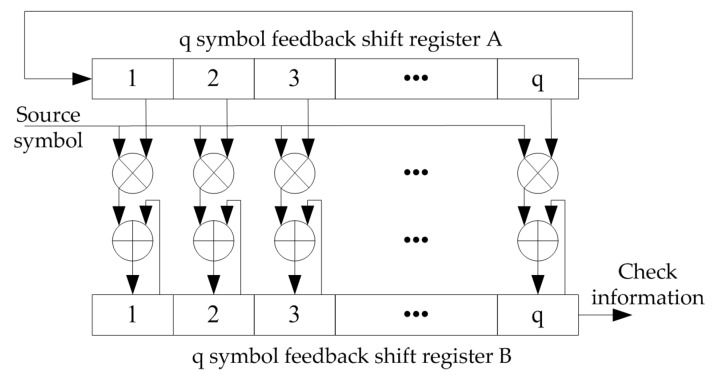
Serial shift register accumulator unit.

The encoder of the QC-LDPC code can be completed by the SSRAA circuit, and its implementation structure is shown in Figure 9.

**Figure 10:**
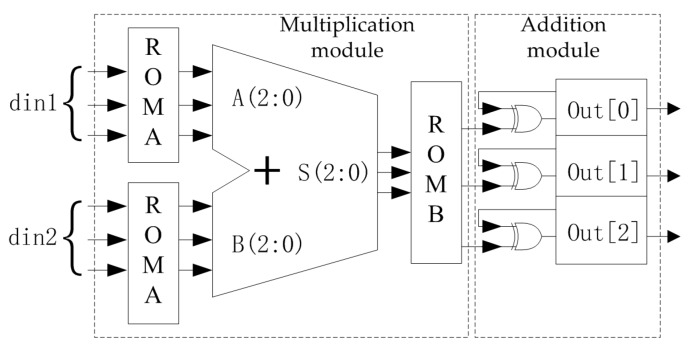
8-ary LDPC code coding unit.

The multiplication and addition operations in the encoding of the NB-LDPC code are operations of the Galois field. For an 8-ary LDPC code, each symbol consists of three bits. The addition of the Galois field is essentially a bitwise XOR operation, as shown in the right part of Figure 10. The multiplication of the Galois field is designed by the looking-up table method, as shown in the left part of Figure 10. First, the input data din1 and din2 are respectively looked up in the ROMA, the result is added in the eight-element domain, and the addition result is checked in the ROMB. The result is the multiplication of din1 and din2 in GF(8).

### 2.4. Complexity Analysis

The coding complexity is shown in Table 1. Since the check matrix construction algorithm for constructing the QC-LDPC code based on the masking and puncturing technique has a quasi-cyclic structure, an encoding method of a quasi-cyclic code with low coding complexity can be employed. From the perspective of coding complexity, adopting the QC-encoding algorithm, only needs to multiply $O\left({\left(L-J\right)}^{2}p\right)$ times in GF(q), and there is no need to add operations throughout the coding process—J and L are the number of rows and columns of the mask matrix $H_{m}$, respectively, and p is the dimension of the sub matrix. Linear complexity coding is achieved when high-speed coding is employed. The coding complexity calculation of the random construction algorithm of the NB-LDPC code check matrix consists of two parts: one is to Gaussian eliminate the check matrix into the structure of the lower triangular matrix, that is, pre-process the check matrix; the operation complexity is $O(n^{3})$, where n is the code length. The other part is that the coding complexity is $O(n^{2})$. This is because the coding complexity of the coding algorithm depends on the sparsity of the generator matrix. Let the average column weight of the generator matrix be w; approximately, wn multiplication operations and (w−1)n sub-operations in GF(q) are required throughout the encoding process. Although the check matrix of a NB-LDPC code is very sparse, its generator matrix is not sparse. Usually, the ratio of w and n is a non-negligible constant, which often makes the coding complexity proportional to the square of its code length. From the hardware storage capacity of the check matrix, when storing the matrix with this construction algorithm, only one p×p unit array I, one J×L non-binary coefficient matrix $Gc_{J\times L}$, and one J×L circulation coefficient matrix $S_{J\times L}$ are stored. However, to construct a parity check matrix of the same size using a random construction method, it is necessary to store a p×J×p×L check matrix. The hardware storage resource is largely saved compared with the storage of the complete NB-LDPC code check matrix.

In addition, the masking and puncturing techniques are used to overcome the shortcomings of the search time when the NB-LDPC code of the long code length is randomly constructed. This is because the dimension of the masking matrix $H_{m}$ is a small constant compared to a randomly constructed matrix. It is easy to construct a check matrix of the NB-LDPC code of the given degree distribution, λ(x) and ρ(x), with the row weight and the column weight.

In the rate-adaptive communication system, the NB-LDPC code of the appropriate code rate is selected according to the channel environment for encoding. Most of the actual communication systems require real-time performance. Improving the effectiveness of the communication system and the speed of hardware processing are two effective ways. With adaptive-rate technology, the average effectiveness of the communication system can be improved. The puncturing of the masking matrix can greatly improve the speed of the hardware processing. Let the masking matrix $H_{m}$ be a binary matrix of J×L and the matrix H be a non-binary matrix of m×n, where m=p×J,n=p×L. For example, if J=8,L=16, the mother code rate is r=1/2, and the puncturing code rate is $r_{m}=3/4$. To delete the check bits in H, 4×p check bits need to be deleted. However, for the check matrix constructed by the algorithm based on masking and puncturing techniques, only 4 check bits of the masking matrix $H_{m}$ need to be deleted. The processing speed of the hardware is increased by p times. Obviously, the longer the codeword, that is, the larger the p value, the more the processing speed of the hardware is improved, thereby greatly improving the real-time performance of the communication system.

## 3. Channel Clustering Based on the K-Means++ Algorithm

Wireless signals are susceptible to the effect of a variety of physical factors during transmission, such as signal source position, obstacles, antenna gain, transmit power, and so on. In order to ensure the validity and reliability of information transmission, it is necessary to change the transmission code rate according to different channel conditions. We usually use channel estimation techniques to judge the quality of channel states. However, the channel estimation algorithm with computational complexity would reduce the effectiveness of the communication system.

With the rise of machine learning, more simple and effective methods are used to reduce the complexity [6,31,32]. The clustering algorithm is an important unsupervised learning algorithm in machine learning. Because of its flexibility, rapid classifications based on different similarities of data are widely used in various technical fields [47,48].

The partition-based clustering algorithm is one of the most commonly used algorithms. Usually, the method based on distance division is used to divide the objects with approximate characteristics into the same cluster. The distances of objects in the same cluster are as close as possible. The distance between objects in different clusters is as far as possible. Partition-based clustering algorithms often need to enumerate the possibilities of all categories in order to obtain an optimal solution.

### 3.1. Channel Environment Clustering Based on the K-Means++ Algorithm

The k-means algorithm is one of the most common partition-based clustering algorithms. K-means clustering aims to partition n observations into k clusters in which each observation belongs to the cluster with the nearest mean, serving as a prototype of the cluster. The k-means algorithm is simple and flexible, but sensitive to outliers and isolated points.

In addition to the impact of the physical factors, the number of users under the same hotspot, as well as the user usage behavior (occupied bandwidth), will have an impact on the actual available channel state. In this paper, we abstract these influencing factors into data vectors and form the data vectors into a sample set, and then cluster them with the k-means algorithm.

We assume that the inputs are the sample set $D=\left\{x_{1},x_{2},\ldots ,x_{m}\right\}$, the cluster number k, the maximum number of iterations N, and the output is cluster partition $C=\left\{C_{1},C_{2},\cdots C_{k}\right\}$. The k-means algorithm flow is as follows:

1. We randomly select k samples from data set D as the initial cluster center vector $\left\{\mu_{1},\mu_{2,},\cdots \mu_{k}\right\}$.
2. For n=1,2,⋯,N
  (a) Initialize cluster partition C to $C_{t}=\emptyset ,t=1,2,\cdots k$.
  (b) For i=1,2,⋯,m,j=1,2,⋯,k, calculate the distance between the sample $x_{i}$ and cluster center vector $\mu_{j}$: $d_{ij}={\left\|x_{i}-\mu_{j}\right\|}^{2}$. Mark $x_{i}$ as the category $\lambda_{i}$ corresponding to the smallest $d_{ij}$ and update $C_{\lambda_{i}}=C_{\lambda_{i}}\cup \left\{x_{i}\right\}$.
  (c) For j=1,2,⋯,k, recalculate the new cluster center for all sample points in $C_{j}$ by $\mu_{j}=\frac{1}{\left|C_{j}\right|}\sum_{x\in C_{j}}x$.
  (d) If all cluster center vectors have not changed, go to step 3.
3. Output cluster division $C=\left\{C_{1},C_{2},\cdots C_{k}\right\}$.

The k-means algorithm is sensitive to the initially selected cluster center, and the position selection of the k initial cluster centers has a great influence on the final clustering result. Therefore, it is necessary to select the appropriate k cluster centers. The k-means++ algorithm randomly initializes the cluster center, which is the optimization of the k-means algorithm.

The optimization strategy of k-means++ for initializing the cluster center is as follows:

According to the process of the algorithm, different initial clustering centers may result in different results. In this paper, we select the k-means++ algorithm in order to optimize the initial clustering center. The k-means++ algorithm is an improvement of the k-means algorithm. Try to choose the k-measuring point distance as the initial cluster center. The specific steps are as follows:

1. Randomly select sample data from the data set as the first cluster center $\mu_{1}$ in k clusters.
2. Calculate the distance D(x) between the data sample $x_{i}$ in the dataset and the nearest cluster center.
  $$D\left(x\right)=\arg\min{\left\|x_{i}-\mu_{r}\right\|}^{2},r=1,2,\cdots ,k_{selected}$$
3. According to the value of D(x), select a new data point in the remaining sample data as a new cluster center. The larger the D(x), the greater the probability of being selected;
4. Repeat steps 2 and 3 until k cluster centers are selected;
5. Implement the standard k-means algorithm by using the k centers as the initial cluster centers.

### 3.2. Complexity Optimization of the End-to-End Distortion Model Based on the Clustering Algorithm

Traditional rate allocation schemes often need to traverse all combinations of coding parameters to achieve adaptive-rate allocation by iterating all combinations. In this paper, the clustering algorithm is introduced to quickly cluster the channel environment to obtain the channel-coding scheme, so as to achieve the goal of adaptive-rate allocation. Compared with the traditional scheme, the proposed method can effectively reduce the computational complexity.

**Figure 11:**
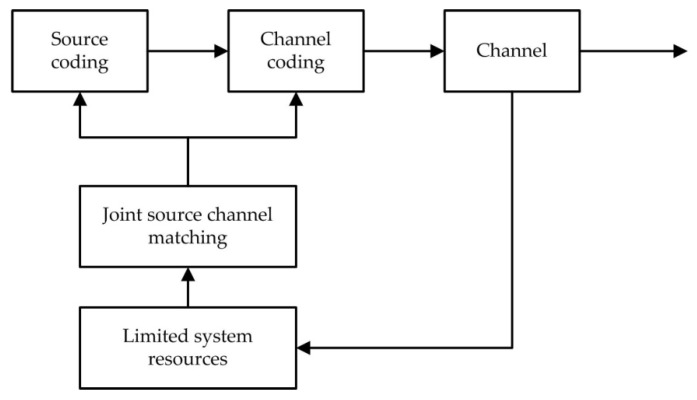
The bit allocation strategy for joint source channel coding.

The joint source channel-coding design based on rate allocation is to obtain the optimal video reconstruction quality through bit allocation between the channel and the source in the case of limited bandwidth and channel interference. A block diagram of the bit allocation strategy for joint source channel coding is shown in Figure 11.

In the joint source channel-coding system, end-to-end distortion is one of the important means to measure the quality of the system. The performance of the transmission system is measured by calculating the mean square error between the reconstructed video image and the original video image at the encoder end. The end-to-end distortion model is derived below.

First, define the pixel in the $n^{th}$ frame as i, and the pixel of the reference frame ref is j. Let $f_{n}^{i}$ be the original value of the pixel, let ${\hat{f}}_{n}^{i}$ and ${\tilde{f}}_{n}^{i}$ be the reconstructed values of the encoder and decoder, respectively, and let ${\hat{r}}_{n}^{i}$ be the reconstructed residual of the encoder, i.e., ${\hat{f}}_{n}^{i}={\hat{f}}_{ref}^{i}+{\hat{r}}_{n}^{i}$. If the current pixel is lost at the decoder side, it will copy the pixels of the ${\left(n-1\right)}^{th}$ frame. Assuming that the transmission error rate is p, the pixel value of the reconstructed image can be expressed by:

(7) $${\tilde{f}}_{n}^{i}=\left\{ \begin{array}{ll} {\tilde{f}}_{ref}^{j}+{\hat{r}}_{n}^{i} & 1-p\\ {\tilde{f}}_{n-1}^{k} & p \end{array}\right.$$

Therefore, the estimated end-to-end distortion of the decoder can be expressed as:

(8) $$\begin{array}{ll} d\left(n,i\right) & =E\left\{{\left(f_{n}^{i}-{\tilde{f}}_{n}^{i}\right)}^{2}\right\}\\ & =\left(1-p\right)E\left\{{\left(f_{n}^{i}-\left({\tilde{f}}_{ref}^{j}+{\hat{r}}_{n}^{i}\right)\right)}^{2}\right\}+pE\left\{{\left(f_{n}^{i}-{\tilde{f}}_{n-1}^{k}\right)}^{2}\right\}\\ & =\left(1-p\right)E\left\{{\left(f_{n}^{i}-{\hat{f}}_{n}^{i}\right)}^{2}\right\}+\left(1-p\right)E\left\{{\left({\hat{f}}_{ref}^{j}-{\tilde{f}}_{ref}^{j}\right)}^{2}\right\}+pE\left\{{\left(f_{n}^{i}-{\tilde{f}}_{n-1}^{k}\right)}^{2}\right\}\\ & =\left(1-p\right)d_{s}\left(n,i\right)+\left(1-p\right)d_{ep}\left(ref,j\right)+pd_{ec}\left(n,i\right) \end{array}$$

It can be seen that the end-to-end distortion can be represented by the sum of the three items $d_{s}\left(n,i\right)$, $d_{ep}\left(ref,j\right)$ and $d_{ec}\left(n,i\right)$, where $d_{s}\left(n,i\right)$ represents source distortion, $d_{ep}\left(ref,j\right)$ represents error propagation distortion from the reference frame, and $d_{ec}\left(n,i\right)$ represents error concealment distortion. Error propagation distortion refers to the use of predictive coding in HEVC coding. If an error occurs in a reference frame or a referenced module, the distortion is propagated to the corresponding frame or pixel with reference to it. Error concealment refers to the distortion caused by the recovery of errors or lost modules generated by transmissions in the channel using temporal or spatial correlation. Usually, in the time domain, the motion-compensated content of the previous frame is used to replace the lost portion of the current frame, and the spatial correlation is used to recover the lost portion in the spatial domain.

Since $d_{s}\left(n,i\right)$ can be obtained by source-encoded parameters at the encoder side, end-to-end distortion mainly depends on the calculation of $d_{ep}\left(ref,j\right)$ and $d_{ec}\left(n,i\right)$. The result shown in Equation (9) can be obtained by calculating $d_{ec}\left(n,i\right)$:

(9) $$\begin{array}{ll} d_{ec}\left(n,i\right) & =E\left\{{\left(f_{n}^{i}-{\tilde{f}}_{n-1}^{k}\right)}^{2}\right\}\\ & =E\left\{{\left(f_{n}^{i}-{\hat{f}}_{n-1}^{k}+{\hat{f}}_{n-1}^{k}-{\tilde{f}}_{n-1}^{k}\right)}^{2}\right\}\\ & =E\left\{{\left(f_{n}^{i}-{\hat{f}}_{n-1}^{k}\right)}^{2}\right\}+E\left\{{\left({\hat{f}}_{n-1}^{k}-{\tilde{f}}_{n-1}^{k}\right)}^{2}\right\}\\ & =d_{ec\_o}\left(n,i\right)+d_{ep}\left(n-1,k\right) \end{array}$$

As can be seen from the above formula, $d_{ec}\left(n,i\right)$ consists of two items, $d_{ec\_o}\left(n,i\right)$ and $d_{ep}\left(n-1,k\right)$, where $d_{ec\_o}\left(n,i\right)$ represents the mean square error between the original image and the encoder-side error-coded pixels, called the original error concealment distortion. $d_{ec\_o}\left(n,i\right)$ can also be obtained by parameter calculation on the encoder side.

It can be seen that $d_{ep}\left(n-1,k\right)$ in Equation (9) and $d_{ep}\left(ref,j\right)$ in Equation (8) represent error propagation distortion. The calculation method of $d_{ep}\left(n,i\right)$ is as shown in:

(10) $$\begin{array}{ll} d_{ep}\left(n,i\right) & =E\left\{{\left({\hat{f}}_{n}^{i}-{\tilde{f}}_{n}^{i}\right)}^{2}\right\}\\ & =\left(1-p\right)E\left\{{\left({\hat{f}}_{n}^{i}-\left({\tilde{f}}_{ref}^{i}+{\hat{r}}_{n}^{i}\right)\right)}^{2}\right\}+pE\left\{{\left({\hat{f}}_{n}^{i}-{\tilde{f}}_{n-1}^{k}\right)}^{2}\right\}\\ & =\left(1-p\right)E\left\{{\left({\hat{f}}_{ref}^{j}-{\tilde{f}}_{ref}^{j}\right)}^{2}\right\}+pE\left\{{\left({\hat{f}}_{n}^{i}-{\hat{f}}_{n-1}^{k}+{\hat{f}}_{n-1}^{k}-{\tilde{f}}_{n-1}^{k}\right)}^{2}\right\}\\ & =\left(1-p\right)E\left\{{\left({\hat{f}}_{ref}^{j}-{\tilde{f}}_{ref}^{j}\right)}^{2}\right\}+pE\left\{{\left({\hat{f}}_{n}^{i}-{\hat{f}}_{n-1}^{k}\right)}^{2}\right\}+pE\left\{{\left({\hat{f}}_{n-1}^{k}-{\tilde{f}}_{n-1}^{k}\right)}^{2}\right\}\\ & =\left(1-p\right)d_{ep}\left(ref,j\right)+pd_{ec\_r}\left(n,i\right)+pd_{ep}\left(n-1,k\right) \end{array}$$

It can be seen that $d_{ep}\left(n,i\right)$ consists of three parts, where $d_{ec\_r}\left(n,i\right)$ represents the reconstructed image and the error concealment distortion at the encoder end, which is called reconstructed error concealment distortion. The expression $d_{ec\_r}\left(n,i\right)$ can be obtained by parameter calculation at the decoder end. Therefore, A is mainly generated by the error propagation distortion of its reference frame and the previous frame. In addition, since the first frame in the video sequence is usually an intra-coded frame, the value $d_{ep}$ of the first frame can be directly obtained without considering error propagation and the values $d_{ep}s$ of the remaining frames can be recursively calculated by Equation (10) on a frame-by-frame basis. In the end-to-end distortion model, the end-to-end distortion of the current frame is obtained by referring to the error propagation distortion of the previous frame, and the subsequent frame is updated by the error propagation distortion of the current frame.

Therefore, from the above analysis, the end-to-end distortion estimation is related to the selection of source-coding, channel-coding, and reference frames.

According to the derivation of the end-to-end distortion estimation method, in the JSCC algorithm based on the rate allocation, in order to select the optimal rate allocation strategy, it is necessary to comprehensively consider the parameters of the source, the channel and the like, so it will be introduced. There is a large number of iterative calculations, so how to choose an effective means to reduce the computational complexity is also a problem worth studying.

Since the end-to-end distortion of the video transmission system is related to the source-coding parameters, the channel-coding parameters, and the reference frame, let S denote the set of all source-coding options, C denote the set of all channel-coding options, and M denote the set of all reference frames. The candidate parameters of the source-coding, channel-coding, and reference frame are u,r,ref, respectively. A system description that minimizes distortion at the decoder is shown in:

(11) $$\underset{u\in S,r\in C,ref\in M}{\min}E\left[d\left(u,r,ref\right)\right]$$

The traditional JSCC algorithm is based on the principle of minimizing end-to-end distortion as a scheme for selecting a code rate allocation. It is often necessary to traverse all combinations of coding parameters, and the worst case computational complexity is O(|S|×|C|×|M|). However, the JSCC scheme based on rate allocation proposed in this paper divides the selection method of the parameter combination into two steps. Firstly, the channel environment is estimated by the clustering algorithm, that is, the code rate r(k) of the channel coding can be obtained by simple clustering before encoding, and then the obtained channel-coding parameters are brought into the minimum end-to-end distortion estimation to obtain the source-coding parameters and the choice of the best reference frame. In the worst case, the computational complexity is O(|S|×|M|). In addition, in practice, since not all of the source-coding options can be used in any of the FCC coding modes of the channel-coding candidate set C, the computational complexity may be even lower.

## 4. Design and Performance Simulation of the Adaptive RC-NB-LDPC Coding Scheme

This section presents the simulation results, which are described in three parts. The first part presents the performance comparison of the constructed RC-NB-LDPC codes with the DVB-S2 short codes. The second part presents the clustering results by the k-mean++ algorithm. The third part presents the advantages of the adaptive coding scheme in compressed image processing.

### 4.1. Design, Analysis, and Simulation of RC-NB-LDPC Codes

The multi-rate LDPC scheme of DVB-S2 is a typical paradigm of the LDPC codes applied in communication standards, which is designed with full consideration of business diversity requirements and has good adaptability. It supports 11 code rates from 1/4 to 9/10, in both normal and short FEC frame modes. The use of ACM is another significant feature of DVB-S2, which provides more accurate channel protection and dynamic connection adaptability for specific receiving terminals according to specific propagation conditions. While improving the original channel transmission capacity, it greatly expands the service range and directly improves network coverage and the user transmission rate. This provides a reference for the ultimate user experience of B5G in the future.

In this section, we first discuss the construction method of RC-NB-LDPC codes, and some parameters such as code length and code rate all refer to the DVB-S2 standard [49]. In addition, we analyze the decoding threshold of the LDPC code by using the EXIT chart [50]. Finally, we simulate the BER performance of the proposed LDPC codes.

#### 4.1.1. Design of RC-NB-LDPC Codes

The LDPC code in the DVB-S2 standard has two kinds of code length, 64800-bit long code, and 16200-bit short-code, and 11 and 10 code rates are specified for long-code and short-code, respectively. The information bit length, check bit length and code-word length of the LDPC code in the DVB-S2 standard are fixed and cannot be arbitrarily selected. Regardless of long-code or short-code, they have the same code-word length and different information n bit length, respectively. Table 2 shows the specific parameters of six typical rates of short-code in the DVB-S2 standard.

**Figure 12:**
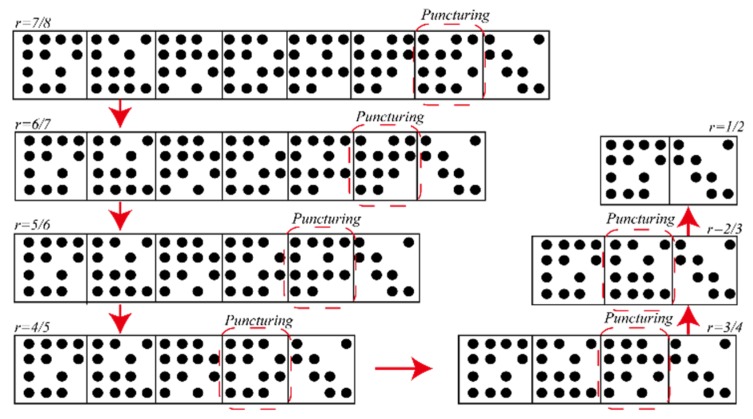
RC-LDPC base matrix scatter diagram.

Through density evolution optimization analysis, the distribution of variable node degrees for all rate designs is (2, 3, 4), which can achieve excellent waterfall performance. On the finite fields, the designed mother matrix rate is 7/8, the size is 4×32, and the expansion factor is p=127. The RCNBQC-LDPC code matrix is designed based on the concealment puncturing technique, and other sub-rate matrixes are obtained from the mother matrix. The particular design is shown in Figure 12. Each time the four columns of the matrix of the current rate are deleted, the number of columns at the same time as 2, 3, and 4 is reduced by 1, 2, and 1. The above process makes the designed matrix of each rate have a superior degree distribution, and the structure is similar, so that codec is simple to implement. The specific parameters of the designed code are shown in Table 3.

#### 4.1.2. EXIT Chart Analysis of RC-NB-LDPC Codes

The Extrinsic-Information-Transfer (EXIT) chart technique is a graphical tool for estimating the decoding threshold of the LDPC code, which is based on Gaussian approximation and can provide intuitive information for the dynamics and convergence of iterative decoding [50]. The EXIT chart is based on the principle that Variable-Node Processors (VNPs) and Check-Node Processors (CNPs) work together and iteratively make bit decisions, with each half-iteration improving the performance metrics. For VNP and CNP, we can obtain the transfer curve of the input metrics and output metrics. The transfer curve of VNP depends on the SNR of the channel. Since the output metric of a processing unit is the input metric of its neighboring processing unit, we can draw two transfer curves in the same coordinate system, with the abscissa and ordinate of a processing unit being exchanged. Such a graph can help predict the code set and decoding threshold for a given variable node and check node degree distribution: the decoding threshold is the signal-to-noise ratio at the focal point of the VNP and CNP transfer curves, which impede the convergence of the two processing units.

The LDPC decoder can be thought of as a bipartite graph. Its parity relationship corresponds to the check nodes. The transmission code-word corresponds to the left n variable nodes. If there is an edge between the i check node and the j variable node in the bipartite graph, the element $H_{i,j}$ in the corresponding check matrix is a non-zero element. The decoding process of the LDPC code is as follows: first, the channel information is used to calculate the initialization information, and then the information between the check node and the variable node is iteratively updated until the information is successfully decoded or the algorithm reached the maximum number of iterations.

**Figure 13:**
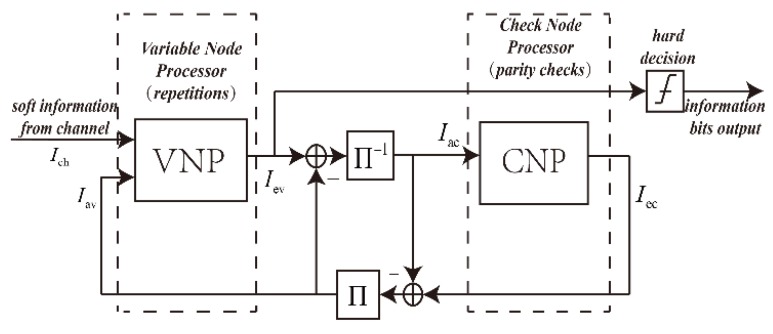
Block diagram of the LDPC decode algorithm.

As is shown in Figure 13, the mutual information amount of the channel message is represented by $I_{ch}$, the input mutual information amount of VND is represented by $I_{av}$, the output mutual information amount is represented by $I_{ev}$, the input mutual information amount of CND is represented by $I_{ac}$, and the output mutual information amount is represented by $I_{ec}$.

We assume that the degree distribution is $\left(d_{v},d_{c}\right)$, while a regular LDPC code rate is $R=1-d_{v}/d_{c}$. When the noise variance is δ (corresponding SNR is Eb/N0) on the AWGN channel, the two EXIT functions are:

(12) $$I_{ev}=J\left(\sqrt{\left(d_{v}-1\right){\left[J^{-1}(I_{av})\right]}^{2}+\delta^{2}}\right)$$

(13) $$I_{ec}=1-J\left(\sqrt{\left(d_{c}-1\right){\left[J^{-1}\left(1-I_{ac}\right)\right]}^{2}}\right)$$

For irregular LDPC codes, $I_{\mathrm{ev}}$ and $I_{\mathrm{ac}}$ are calculated based on the weighted average. The weights are given by the coefficients of the degree distribution polynomials λ(X) and ρ(X) ($\lambda \left(X\right)=\sum_{d=1}^{d_{v}}\lambda_{d}X^{d-1}$ and $\rho \left(X\right)=\sum_{d=1}^{d_{c}}\rho_{d}X^{d-1}$), where λ is the proportion of edges in the Tanner graph connected to a d degree variable node, and ρ is the proportion of edges connected to a d degree check node. Moreover, λ(1)=ρ(1)=1. Therefore, for irregular LDPC codes:

(14) $$I_{ev}=\sum_{d=1}^{d_{v}}\lambda_{d}I_{ev}\left(d,I_{av}\right)$$

where $I_{ev}\left(d\right)$ is given by (14) and just $d_{v}$ needed to be replaced in the formula with d.

(15) $$I_{ec}=\sum_{d=1}^{d_{c}}\rho_{d}I_{ec}\left(d,I_{ac}\right)$$

where $I_{ec}\left(d\right)$ is given by (15) and just $d_{c}$ needed to be replaced in the formula with d.

**Figure 14:**
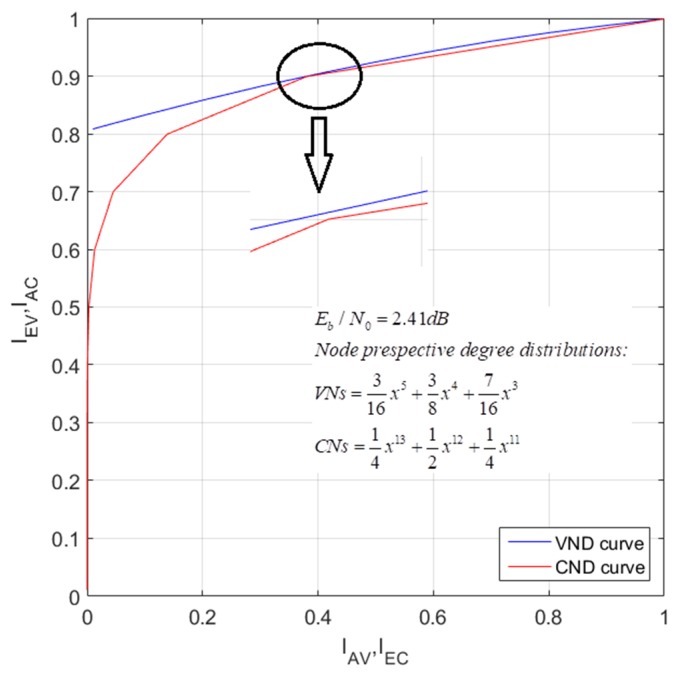
EXIT chart of the designed degree distributions.

These equations can be used to calculate the EXIT curves for VND and CND. For a given SNR and a set of degree distributions, increase the SNR if the two curves intersect. Otherwise, the SNR would be decreased. The SNR that makes them exactly intersect is the decoding threshold. Figure 14 shows the EXIT curve of the designed LDPC code when the rate is 3/4. It can be seen that when *E_b_*/*N*_0_ = 2.41 dB, the two curves do not intersect, indicating that the decoder converged and the decoding threshold is 2.41 dB. The decoding thresholds of the other rates of the designed LDPC codes and the DVB-S2 short-code are compared in Table 4. It can be clearly seen that the designed LDPC codes are better than DVB-S2 at each rate.

#### 4.1.3. Simulation of RC-NB-LDPC Codes

This section performs performance simulation and analysis of the RC-NB-LDPC code. The parameters are as follows: the decoding algorithm is a Mixed-LOG-FFT-BP decoding algorithm, the maximum number of iterations is 20, the modulation mode is BPSK, and the channel is an AWGN channel. Monte Carlo simulation was performed and the following results were obtained.

**Figure 15:**
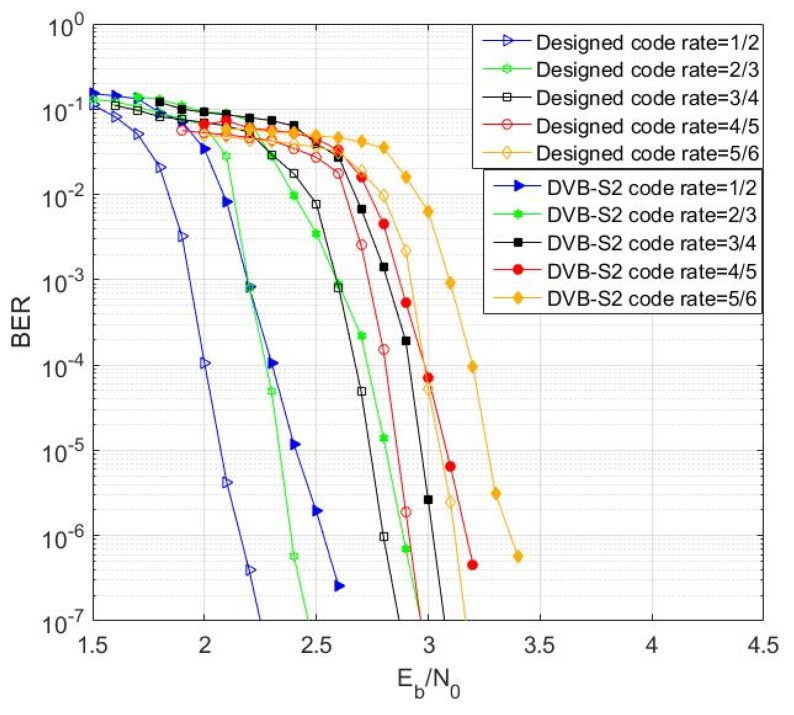
BER simulation comparison result.

**Figure 16:**
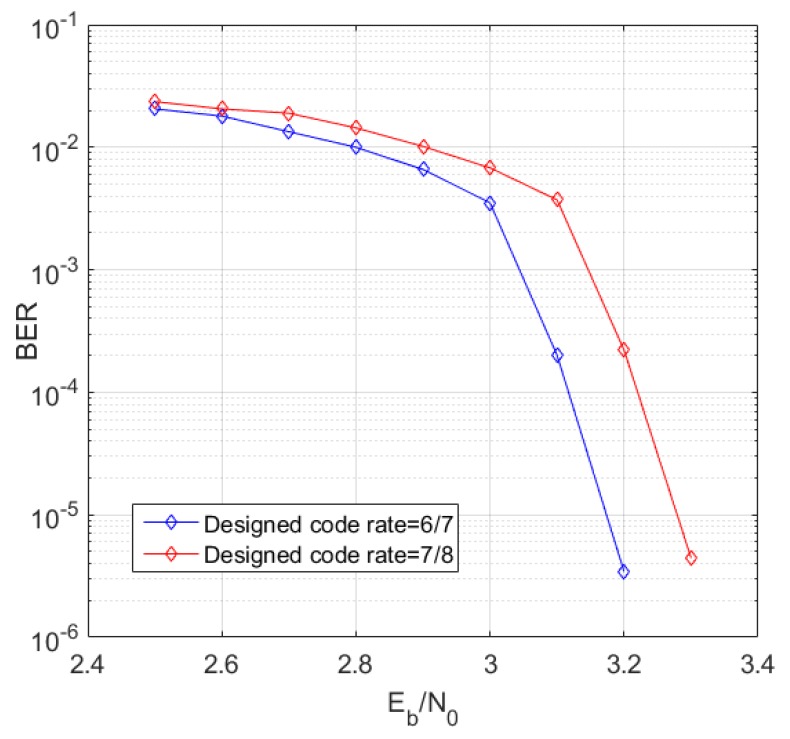
Designed 7/8 rate code performance.

Figure 15 and Figure 16 simulate the bit error rate (BER) performance of the RC-NB-LDPC codes with different code rates. As can be seen from the figure, the constructed LDPC code achieves better waterfall performance in a larger code rate range. Moreover, the constructed RC-NB-LDPC code achieves a performance gain of about 0.2–0.5 dB compared to the LDPC code in the DVB-S2 standard.

**Figure 17:**
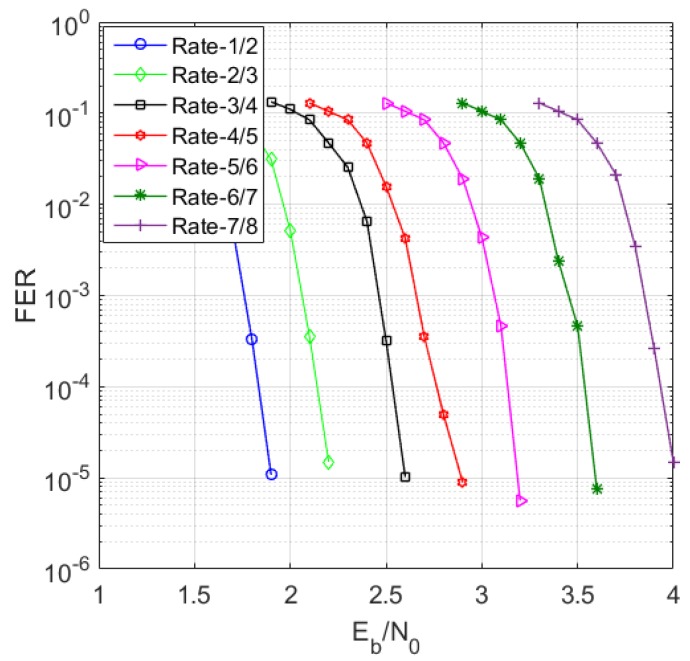
FER simulation result.

Figure 17 simulates the frame error rate (FER) performance of the RC-NB-LDPC codes with different code rates. It can be seen that the RC-NB-LDPC code achieves better anti-interference performance in a larger code rate range.

**Figure 18:**
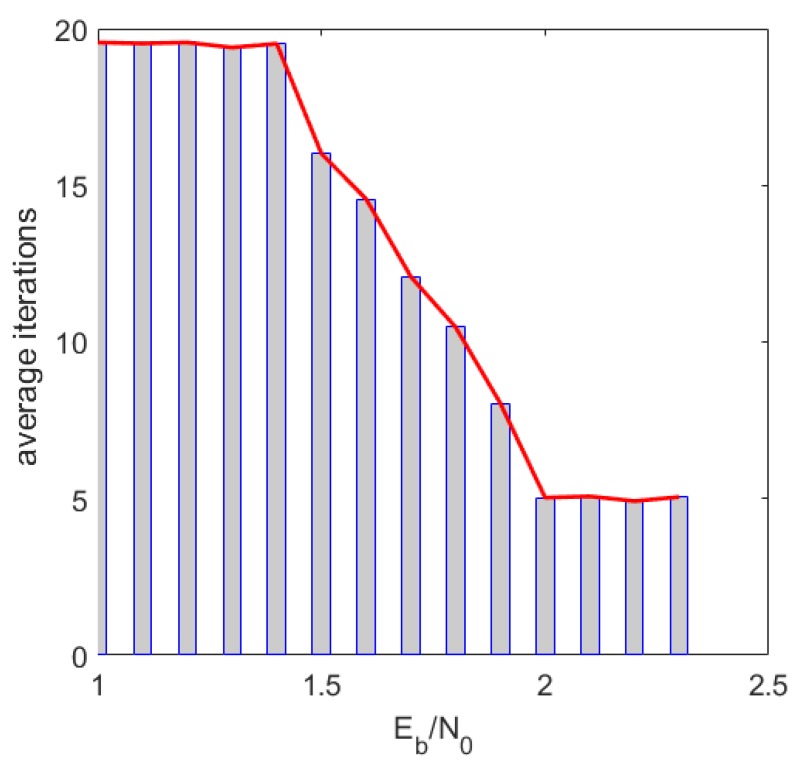
Average iterations simulation result at R = 0.5.

Figure 18 simulates the average number of iterations of the RC-NB-LDPC code with a code rate of 1/2. As can be seen from the figure, the higher the $E_{b}/N_{0}$, the smaller the average number of iterations. In addition, the RC-NB-LDPC codes have low decoding delay and are suitable for real-time communication scenarios.

### 4.2. Simulation of the Channel Clustering Algorithm

In this section, we combine the physical factors that affect the state of the wireless channel with the channel bandwidth to form a training sample. The physical factors in the wireless channel are represented by the signal-to-noise ratio (SNR), and the value range is [0, 4.5] dB. The transmission bandwidth is [1000, 8000] KHz. The training sample data set consists of a data set that conforms to a Gaussian distribution and contains 2000 data points. The k-means++ algorithm is used to cluster data, and k=3.The clustering result is shown in Figure 10.

**Figure 19:**
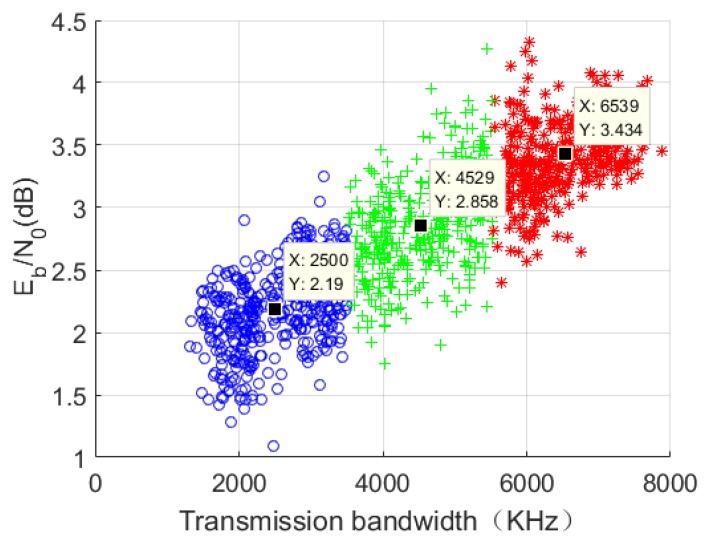
Clustering of simulated wireless channel conditions.

The data points with different symbols in Figure 19 indicate different categories. It can be seen that a series of unordered channel data is divided into three categories by the clustering algorithm.

### 4.3. Design and Performance Simulation of the Adaptive RC-LDPC Coding Scheme

#### 4.3.1. The Design of the Adaptive RC-LDPC Coding Scheme

**Figure 20:**
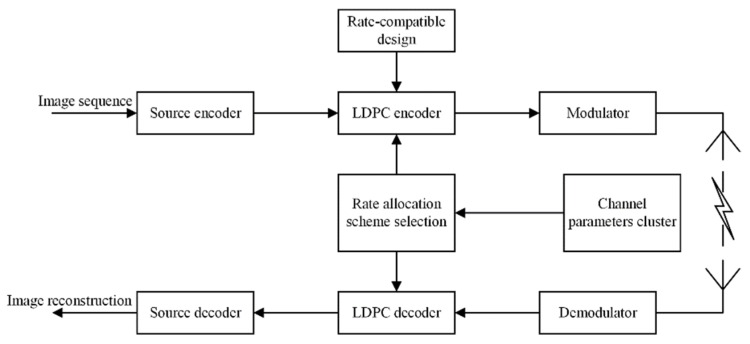
The adaptive RC-LDPC coding scheme block diagram.

In this section, we take the image transmission as an example, and propose a rate-compatible LDPC coding scheme based on channel clustering, as shown in Figure 20. The basic process of the program is divided into three parts:

1. Channel clustering

Using the data set composed of different channel environment parameters and user usage behavior as training data, the k-means++ algorithm is used to divide the wireless channel situation into k different classes.

2. Rate allocation

We use the set partitioning in hierarchical trees (SPIHT) algorithm as the image compression algorithm. The SPIHT algorithm has extremely low computational complexity and high-quality recovery characteristic and breaks the boundary between coding efficiency and complexity in traditional coding algorithms [51]. It makes reasonable use of multi-resolution characteristics after wavelet decomposition and has good coding performance. The higher compression rate causes the output code stream to be very sensitive to channel errors. Therefore, the worst one of each channel class is selected as the basis for designing the code rate allocation, ensuring that under all channel conditions in the channel class, the compressed code stream is transmitted as accurately as possible. A corresponding code rate allocation scheme is designed according to the worst channel condition in each cluster.

3. Encode and update the cluster center

When the image is transmitted in the wireless channel, the code rate allocation scheme is adaptively adjusted by the clustering of the current channel data, and the corresponding source and channel coding are performed by using the code rate allocation scheme. Finally, the real-time data of the channel at this time is used as training data to update the cluster center.

#### 4.3.2. Simulation Results and Analysis

1. Image quality evaluation

We evaluate the performance of the transmission scheme by comparing the quality of the reconstructed image with the original image. The evaluation methods include subjective evaluation and objective evaluation.

Subjective evaluation is a direct and important evaluation method for subjective judgment by observing and comparing the difference between the reconstructed image and the original image. However, in practice, individual differences in observers are likely to cause subjective bias. Moreover, subjective evaluation often requires many labor and material resources, and it is impossible to perform real-time detection.

The objective evaluation method usually uses the error between the reconstructed image and the original image to measure the quality of the reconstructed image. Common evaluation methods include Mean Squared Error (MSE) and Peak Signal Noise Ratio (PSNR) [51]. The calculation formula for MSE is:

(16) $$\mathrm{MSE}=\frac{\sum_{i=1}^{M}\sum_{j=1}^{N}{\left(f\left(i,j\right)-f{\left(i,j\right)}^{\prime }\right)}^{2}}{M\times N}$$

where f(i,j) and $f{\left(i,j\right)}^{\prime }$ represent the pixel values of the original image and the reconstructed image at (i,j), respectively, and M×N is the size of the image. MSE reflects the quality of image restoration. It can be seen that the smaller the MSE value, the smaller the deviation between the reconstructed image and the original image, and vice versa. The formula for calculating PSNR is:

(17) $$\mathrm{PSNR}=10\log_{10}\frac{255}{\mathrm{MSE}}$$

PSNR and MSE are essentially the same. The larger the PSNR value, the smaller the deviation between the restored image and the original image, and vice versa. PSNR is often viewed as a quality metric and MSE as a distortion metric.

2. Reconstructed image quality simulation

In this paper, we select the “Lena” 512 × 512 grayscale image as the test image. The source-coding method is the SPIHT algorithm, the channel-coding method is rate-compatible LDPC code, and the channel model is the AWGN channel. We select three different channel conditions as test conditions. The specific parameters of the channel are shown in Table 5.

We performed a performance analysis comparison of the following three transmission schemes:

- Scheme 1, the fixed-rate transmission scheme. That is, the channel coding uses a fixed code rate of 3/4 for three types of test channels in this paper.
- Scheme 2, the adaptive-rate transmission scheme. That is, after channel clustering, the corresponding rate is selected for each type of test channel. Corresponding to three types of test channels, the coding rates used in this paper are 1/2, 4/5, and 7/8, respectively.
- Scheme 3, the adaptive-rate transmission scheme with UEP. According to different parts of the code stream that have different effects on image recovery, we adopt different levels of the channel protection mechanism, that is, the high-level protection for the important code stream and the low-level protection for the secondary code stream. Corresponding to the three types of test channels, the coding rate groups in this paper are (1/2, 3/4), (2/3, 5/6) and (3/4, 7/8), respectively.

The scheme affects the different degrees of the restored image according to different parts of the code stream, adopts different levels of channel protection mechanisms, protects the important code stream to a high degree, and protects the secondary code stream to a low degree to achieve different degrees of differentiation.

In this paper, the PSNR of the reconstructed image is chosen as the criterion for evaluating image quality. The simulation results are shown in Table 6. The “fail” in the table indicates that the decoding is unsuccessful or the reconstructed image quality is extremely poor due to severe error. In order to ensure the correctness of the results, we repeat the transmission process 50 times and take the average result.

As shown in Table 6, with the fixed bit rate scheme, in the case of poor channel conditions, a decoding failure occurs. This is because the fixed bit rate employed does not provide sufficient channel protection under poor conditions. For image compression streams, if the error occurs at a critical location and there is no error retransmission or other means of recovery, it will result in decoding failure or poor reconstructed image quality.

It can also be seen that compared to scheme 1, scheme 2 achieves an average gain of about 2 dB. The reason is that scheme 2 adopts different code rates according to different channel conditions. When the channel condition is poor, low code rate coding is used to enhance channel protection. When the channel environment is good, high code rate coding is used to improve the overall efficiency of the system.

In addition, by comparing the data of scheme 2 and scheme 3, we can also see that since scheme 3 adopts UEP, the reconstructed image quality can be further improved compared with scheme 2, and the PSNR of the reconstructed image can obtain an average of about 0.5 dB.

**Figure 21:**
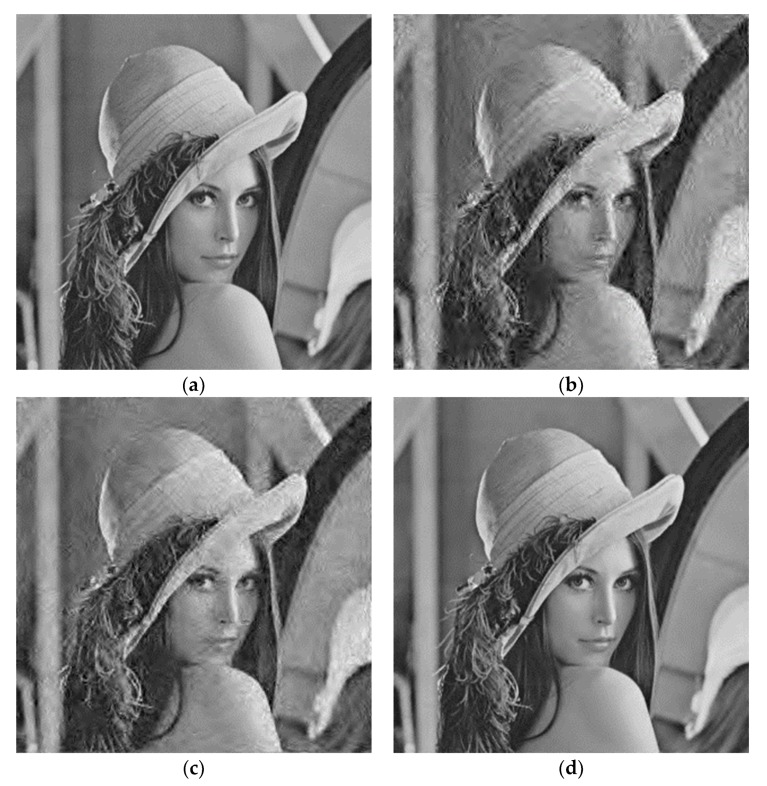
Reconstruction of images under different schemes. (**a**) is the original image, and image (**b**–**d**) are reconstructed images using the three schemes, respectively.

At an image compression ratio of 0.5, Figure 21 shows reconstructed images obtained by using the three different schemes under the conditions of the channel environment of Class 2. Image (a) is the original image, and image (b), image (c) and image (d) are reconstructed images using the three schemes, respectively. We can see that the image quality of image (b), image (c) and image (d) is sequentially improved. Moreover, image (d) and the original image (a) are almost identical.

## 5. Conclusions

For the B5G mobile system, this paper first proposed a construction method of the RC-NB-LDPC code, which is realized by combining masking and puncturing technology. The RC-LDPC code constructed by the method has an easy hardware implementation of the check matrix structure and better waterfall performance in a larger code rate range. Compared with the LDPC codes in the DVB-S2 standard, the RC-NB-LDPC codes achieved a performance gain of about 0.2–0.5 dB, which is suitable for data transmission. Then, according to the influencing factors of the wireless channel, this paper proposed using the k-means++ algorithm to cluster the wireless channel environment to obtain the code rate allocation scheme. Finally, this paper took compressed image transmission as an example, applied the clustering algorithm to the fast classification of channel environment, combined the characteristics of the SPIHT code stream structure, and studied the problem of compressed image transmission in the wireless channel. In the process of transmitting the compressed image code stream, the proposed adaptive-rate transmission scheme can effectively utilize the limited bandwidth resources of the channel to ensure reliable transmission of image information, and obtain higher reconstructed image quality than the fixed code rate-coding scheme. Further enhancements can be obtained by combining the UEP strategy. Through simulation, the adaptive-rate transmission scheme can achieve a performance improvement of about 2 dB over the fixed-rate transmission scheme. Moreover, using the adaptive-rate transmission scheme with UEP, the PSNR of the reconstructed image can be additionally increased by about 0.5 dB on average.

This paper only provides an adaptive channel-coding scheme, but the other advanced source-coding techniques can be used to further design a better joint source channel-coding scheme. In addition, channel-coding technology can be combined with modulation, power control and other technologies to design a more complete adaptive transmission scheme.

## Figures and Tables

**Table 1:** Coding complexity analysis.

Matrix Construction Algorithm	Pre-Process	Add Operation Times in GF(q)	Multiplication Operation Times in GF(q)	Coding Complexity
Random construction	$O(n^{3})$	(w−1)n	wn	$O(n^{2})$
Masking and puncturing construction	No	No	$o\left({\left(L-J\right)}^{2}p\right)$	O(n)

**Table 2:** Parameters of the short codes in the DVB-S2 standard.

Code Rate	Information Bitlength	Code-Word Length	Variable Node Degree	Variable Node Degree Distributions
4/9 (≈1/2)	7200	16,200	(1, 2, 3, 8)	(1, 8999, 5400, 1800)/16,200
2/3	10,800		(1, 2, 3, 13)	(1, 5399, 9720, 1080)/16,200
11/15 (≈3/4)	11,880		(1, 2, 3, 12)	(1, 4319, 11,520, 360)/16,200
7/9 (≈4/5)	12,600		(1, 2, 3)	(1, 3599, 12,600)/16,200
37/45 (≈5/6)	13,320		(1, 2, 3, 13)	(1, 2879, 12,960, 360)/16,200

**Table 3:** Parameters of the designed RC-NB-LDPC codes.

Code Rate	Information Length (Bit/Symbol)	Code Length (Bit/Symbol)	Base Matrix Size	Expansion Factor	Variable Node Degrees	Variable Node Degree Distributions
7/8	14,224/3556	16,256/4064	4 × 32	127	(4, 3, 2)	(7, 14, 11)/32
6/7	13,920/3480	16,240/4060	4 × 28	145	(4, 3, 2)	(6, 12, 10)/28
5/6	13,440/3360	16,128/4032	4 × 24	169	(4, 3, 2)	(5, 10, 9)24
4/5	12,992/3248	16,240/4060	4 × 20	203	(4, 3, 2)	(4, 8, 8)/20
3/4	12,144/3036	16,192/4048	4 × 16	253	(4, 3, 2)	(3, 6, 7)/16
2/3	10,816/2704	16,224/4056	4 × 12	338	(4, 3, 2)	(2, 4, 6)/12
1/2	8096/2024	16,192/4048	4 × 8	506	(4, 3, 2)	(1, 2, 5)/8

**Table 4:** Threshold Predictions of Designed Codes.

Code Rates	Thresholds under AWGN Channel (dB)	
	DVB-S2 Scheme	RC-NB-LDPC Codes
7/8	----	2.87
6/7	----	2.81
5/6	2.86	2.72
4/5	2.70	2.59
3/4	2.58	2.41
2/3	2.38	2.12
1/2	2.02	1.80

**Table 5:** Test channel parameter.

Channel Types	Test Parameters $\left[E_{b}/N_{0},\;\mathrm{Bandwidth}\right]$
Class 1	[2.2, 2500 KHz]
Class 2	[2.8, 4500 KHz]
Class 3	[3.5, 6500 KHz]

**Table 6:** Reconstructed image PSNR for different schemes.

Compression Ratio	Test Schemes	PSNR (dB)		
		Class1	Class2	Class3
1	Scheme 1	Fail	33.53	35.94
	Scheme 2	31.79	35.24	37.68
	Scheme 3	32.38	35.75	38.23
0.5	Scheme 1	Fail	30.71	32.77
	Scheme 2	28.86	32.47	34.64
	Scheme 3	29.33	32.91	35.14
0.1	Scheme 1	Fail	27.82	29.24
	Scheme 2	27.26	29.01	29.32
	Scheme 3	27.68	29.28	29.32
